# Observations on Hooping-Cough

**Published:** 1831

**Authors:** 


### XVIII.

**OBSERVATIONS ON HOOPING-COUGH. By M. BLAND, Physician-in-Chief to the Hospital Beaucaire.**

DURING a severe epidemic hooping-cough in France, the author of this paper suffered an attack of the disease, and was naturally enough led to examine into its nature, causes, and treatment, with more critical scrutiny than a non-professional patient or observer.

The author makes some pertinent observations on the peculiarity of the cough, the sickness, and the cerebral congestion, which we see in pertussis. The cough is remarkable for the number of short and interrupted expirations which succeed to a long and sonorous inspiration. These expirations, which amount to eight or ten in infancy, diminish according to age; and, in the adult, seldom exceed two, three, or four. In the latter cases, too, the inspirations lose a great deal of their sonoriety—circumstances which prove that this symptom and the number of successive expirations, form not the essential characters of hooping-cough. The cough is provoked by a sense of titillation or pricking, more or less sharp, in the place where the trachea divides into the bronchia—the real seat of the disease, according to M. Bland. This titillation is sometimes slow, sometimes rapid. In the *former* case, the patient has a presentiment of the paroxysm, in the shape of inquietude, even some fear—and an eagerness to secure some position that may enable him to breathe more freely during the attack, or rather to expel, with more facility, the expectoration. In the *second* case, the titillation comes on instantaneously, and there is little or no premonitory sensation. In both cases the inspiratory muscles, as well as those of the larynx, are thrown into a kind of convulsive action; hence the profound inspiration to fill every air-cell, and the violent efforts to expel the secreted humour from the lungs. The expectoration, however, is rarely so great in quantity as in cases of common catarrh. When the irritating fluid is not completely expelled by the paroxysm, the titillation continues after the fit; and another attack is soon to be expected. These paroxysms are more frequent after taking food, probably by a propagation of excitement from the gastric to the bronchial mucous membrane. In the course of a few days after the commencement of the cough, and during the whole of its progress, there is felt, at every considerable exertion of the breathing, as, for example, during sneezing or the paroxysm itself, a pain, more or less acute, in the line of the attachments of the diaphragm, resulting, no doubt, from the convulsive action of that muscle in each attack. When the expectorated matter reaches the mouth, it tastes like hydro-chlorate of soda, and seems to excite an inordinate quantity of saliva from the glands of the mouth and fauces. This secretion is so copious and acrid as apparently to excite the vomiting which so often occurs in hooping-cough. This vomiting is much less in adults than in young persons, probably from the sympathy between the two sets of membranes being less acute in the one than in the other.

Our author is of opinion that the symptoms of cerebral turgescence, or even congestion, which are experienced during the paroxysms of hooping-cough, and for some time afterwards, are occasioned by what he terms "the mechanism of the cough," (dépend du mechanisme du toux)—namely, on the afflux of arterial blood to the brain, and the retardation of the venous current from that organ, by the cough and by the pulmonary congestion. The cephalalgia, experienced chiefly in the temples, and the tremor of the upper extremities, are the results of this mechanical turgescence in the cerebral vessels. After the paroxysm, in ordinary circumstances, all the functions come back to their common level of tranquillity.

*Nature of Hooping-Cough.* This has been looked upon by some as a catarrhal affection complicated with spasm—by others, as a cough purely and essentially spasmodic—while some have placed the seat of pertussis in the head, the stomach, the liver, &c. M. Bland confesses that he himself has failed to ascertain the true nature of the disease. He thinks, however, that we may fairly assume that it consists in a morbid secretion of the bronchial mucus—a specific secretion, sui generis—saturated, in some way or other, with hydro-chlorate of soda, the irritation of which excites the paroxysms of coughing. The author's own personal observations, and the statements of others capable of analyzing and appreciating their own sensations, confirm this supposition. He thinks this view of the disease is supported by analogy. Thus various secretions, as the tears, the bile, &c. sometimes become so acrid and vitiated as to excoriate the parts with which they come in contact. He remarks that this peculiar secretion is clear and almost transparent throughout the whole course of the disease, and never takes on the muco-purulent appearance of common catarrhal inflammation. He concludes, therefore, that it is irritation, not inflammation of mucous membrane that we have to deal with. This peculiar and morbid humour, he thinks, is secreted more copiously in the night than in the day—hence the paroxysms are more frequent and violent in the former portion of the 24 hours. When the affection is largely extended throughout the bronchial ramifications, and very intense at the same time, the patient dies of suffocation during the prolonged paroxysms—or in consequence of the cerebral congestion resulting from the violence of the disease.

Our author then asks, what is the cause of this peculiar and acrid secretion in the mucous glands and follicles of the bronchia? Does it arise from atmospheric vicissitudes, or from a peculiar principle floating in the atmosphere? These questions, he confesses, we are unable at present to answer. We think there can be little hesitation in denying that mere atmospherical vicissitudes are the primary cause of hooping-cough. We might just as well conclude that small-pox was the result of aerial transitions.

"However that may be, (says our author,) the treatment which is most rational, which is most safe, is that which brings back the bronchial secretion to a healthy state—but as that treatment is yet unknown, we have a key to the numerous specifics which are cried up as infallible in this disease."

He considers it as proved, however, that belladonna, in powder or extract, exercises a beneficial influence on hooping-cough; but he thinks that a still more powerful agent in mitigating the disease is to be found in the sulphuret of potash; of the efficacy of which he narrates some cases as proofs. We shall glance at some of these.

*Case* 1. G. Pongé, aged 21 years, and affected with hooping-cough for about three weeks, entered the hospital BEAUCAIRE on the 18th January, 1831, for the treatment of the complaint. The paroxysms were violent, and frequent, especially at night. The expectoration had a saline taste. He was ordered *a grain* of the extract of belladonna four times a day. (Surely this must be a mistake; or else the medicine must be very different in strength on the Continent and here.)

During the first ten days there was no sensible alteration. On the 29th of January, ten grains of the sulphuret of potash in honey were given, night and morning. Even in the first 24 hours the paroxysms were mitigated. The medicine was continued. On the 31st the cough is merely catarrhal, and the expectoration is no longer saltish. On the 2d of February the hooping was at an end, and the medicines were discontinued.

*Case* 2. Joseph Pongé, brother of the former, aged 23 years, entered the hospital on the same day as the other patient, 18th January. He had only been four days ill with hooping-cough. The paroxysms were still more violent than those of the brother, producing a considerable cephalic congestion, requiring bleeding from the arm. The belladonna was prescribed, as for the other patient, but no evident amelioration was perceptible. On the 29th January, ten grains of the sulphuret were ordered night and morning. The night was more quiet than usual. 30th and 31st. The same medicine. He had only one paroxysm in the night. The expectoration was less saline. On the 2d of February, he was discharged cured.

*Case* 3. This was a young man, 20 years of age, who entered the hospital on the 20th January, having been sixty days ill with hooping-cough. His face was bloated, and the vessels injected by the paroxysms of coughing, which were violent and frequent. The expectoration was very saline and acrid. The extract of belladonna was given him, as in the former case, but with very little advantage. On the 29th January, ten grains of the sulphuret of potash were ordered twice a day. This was continued on the 30th, 31st, and till the 2d of February, when the patient was only affected with simple catarrhal cough, and was discharged cured.

"We see, says the author, in the three foregoing cases, with what promptitude the sulphuret of potash modified the morbid secretion which constitutes hooping-cough—changed it into simple mucus—and thus stopped the paroxysms of cough which this morbid fluid excited."

Although this is no new remedy, and we are far from being so sanguine as M. Bland as to its efficacy, we have thought it proper to lay the facts before our readers, such as we found them.—REVUE MEDICALE.

